# The impact of a Eurocentric curriculum on racial disparities in maternal health

**DOI:** 10.18332/ejm/140086

**Published:** 2021-09-06

**Authors:** Sarah Esegbona-Adeigbe

**Affiliations:** 1 Adult Nursing and Midwifery Studies, School of Nursing and Midwifery, Institute of Health and Social Care, London South Bank University, London, United Kingdom

**Keywords:** midwifery education, Eurocentric curriculum, racial disparities, maternal morbidity

**Dear Editor,**

Black, Asian and ethnic minority women have a fourfold risk of dying in the UK compared to White women^1^. This racial disparity is mirrored in other European countries, where women of ‘non-western’ origin have a 60% higher rate of maternal mortality^2^. Midwifery education should prepare midwives to meet the needs of ethnically diverse women, but there are concerns about a failure to address the issues of racial disparities in maternity services. It is acknowledged that racial bias leads to poorer health outcomes and experiences for Black, Asian and ethnic minority women by negatively influencing their care and treatment^3^. Midwifery education plays an important role in quality midwifery care and producing midwives that are fit for purpose and fit for practice. Recommendations have been made by the UK Royal College of Obstetricians and Gynaecologists including that being aware of explicit and implicit racial bias should be part of the curriculum and students need to be aware that implicitly held negative stereotypes and beliefs about race, ethnicity and gender influence interactions with individuals and the care that they receive^3^.

To achieve quality midwifery care that reduces racial disparities requires an effective midwifery workforce, an effective curriculum and academic staff who can teach and deliver the curriculum^2^. However, it can be argued that racial disparities caused by racial bias have historical roots that are still present in contemporary healthcare. Colonization of countries is a relic of the past, and, despite this, higher education curricula continue to be colonized by White and Western intellectual traditions with lack of appropriate representation of ethnically diverse groups^4^. Hence, the existence of Eurocentric curricula that are colonized with teaching and learning that do not acknowledge White privilege and the mindsets that have been created and sustained by this^5^. A Eurocentric curriculum that portrays a White, male and heterosexual view does not cater for the needs of women from ethnically diverse backgrounds^4^. The use of a Eurocentric curriculum means that students are expected to engage with content that does not relate to them and taught that racial diversity is a problem and a deficit^5^. Midwifery students and other healthcare professional students should be equipped with the skills and knowledge to care for women from ethnically diverse backgrounds equitably and non judgementally^6^. A failure to teach midwifery students culturally appropriate assessments, failure to acknowledge systemic racism and discrimination, and non diverse teaching staff, all contribute to racial disparities in maternal health. A non-Eurocentric curriculum is engaged, anti-racist and not colonial^7^, and should be inclusive and representative of different communities, voices, and perspectives^4^. Ethnically diverse women face adverse pregnancy outcomes, perpetuated by midwives who have not been given the skills and knowledge to understand their needs and who relegate their concerns as unimportant. To reduce racial disparities in maternal health requires a radical look at the midwifery curriculum to remove inaccurate and negative colonial influences and bring midwifery education into the 21st century.

## Data Availability

Data sharing is not applicable to this article as no new data were created.
